# Assessing Long-Term Liver Safety of Statins and PCSK9 Inhibitors Using Human Genetics

**DOI:** 10.1016/j.jcmgh.2023.10.008

**Published:** 2023-11-06

**Authors:** Marijana Vujkovic

**Affiliations:** Department of Medicine, University of Pennsylvania Perelman School of Medicine, Philadelphia, Pennsylvania; Department of Biostatistics, Epidemiology and Informatics, Perelman School of Medicine, University of Pennsylvania, Philadelphia, Pennsylvania; Corporal Michael J. Crescenz VA Medical Center, Philadelphia, Pennsylvania

Cardiovascular disease is the leading cause of death worldwide, and high low-density lipoprotein (LDL) cholesterol is a major causal risk factor.^1^ The cornerstone of primary and secondary prevention of atherosclerotic cardiovascular disease is intensive lipid-lowering therapy, targeted primarily at reducing LDL cholesterol.^2^ This currently is accomplished with the use of statins, ezetimibe, and, more recently, monoclonal antibodies that inhibit proprotein convertase subtilisin–kexin type 9 (PCSK9).^3^ Statins and PCSK9 inhibitors reduce LDL cholesterol levels by increasing the density of LDL receptors on the surface of liver cells, resulting in the increased binding, uptake, and clearance of apolipoprotein B-100–containing lipoproteins (mainly LDL) from the bloodstream into the liver, while ezetimibe blocks cholesterol uptake in the intestine.

Statins exert their action by inhibiting 3-hydroxy-3-methylglutaryl coenzyme A (HMG-CoA) reductase activity, thus restricting intracellular cholesterol production and triggering liver cells to up-regulate LDL-receptor expression. PCSK9 is responsible for the degradation of LDL receptors in the lysosome, and PCSK9 inhibitors work by either blocking hepatic synthesis of PCSK9 or preventing the binding of circulating PCSK9 to LDL receptors. Because both HMG-CoA and PCSK9 inhibitors increase the exposure of the liver to cholesterol, it has been hypothesized that this might increase the potential for steatosis, a concern akin to what is observed with microsomal triglyceride transfer protein inhibitors (lomitapide), angiopoietin-like 3 inhibitors (evinacumab), and apolipoprotein B-100 messenger RNA antisense oligonucleotide inhibitors (mipomersen).^3^ Fortunately, safety data from randomized clinical trials have shown that statins and PCSK9 inhibitors are well tolerated, with low rates of adverse events, except for mild injection site reactions for PCSK9 inhibitors and muscle pain for statins, but longer-term safety of PCSK9 inhibition remains to be evaluated.^4^^,^^5^

Human genetics played a critical role in the development of PCSK9 inhibitors, driven by the observation that individuals carrying loss-of-function PCSK9 mutations have a lower level of LDL cholesterol and lower rates of cardiovascular events compared with individuals without the mutation.^6^ These naturally occurring differences in cardiovascular outcomes among otherwise comparable individuals, distinguished solely by their randomly inherited PCSK9 allele status, have been likened to nature's own randomized trial of PCSK9 inhibition, or Mendelian randomization.^7^ This observation has since been clinically validated through the observed reductions in cardiovascular disease in randomized trials involving PCSK9 inhibition.^8^

In this issue of *Cellular and Molecular Gastroenterology and Hepatology*, Rosoff et al^9^ present an important study of liver safety of statins and PCSK9 inhibitors using the same approaches that motivated PCSK9 clinical development. They performed a multi-ancestry drug target Mendelian randomization study to examine the relationship between genetic inhibition of PCSK9 and HMG-CoA reductase on circulating markers of liver injury, such as bilirubin and aminotransferases. Using large genetic data sets across 4 ancestries, they proxied ancestry-specific effects of LDL cholesterol, with secondary analyses using data on circulating protein levels and tissue-specific gene expression. As a result, Rosoff et al^9^ discovered no compelling evidence indicating that reduction of LDL cholesterol through the long-term inhibition of PCSK9 or HMG-CoA reductase had any discernible effect on circulating markers of liver injury across all ancestries. These observed nonassociations are unlikely to be attributed to inadequate study power because all analyses had sufficient statistical power to detect effects across all ancestral groups.

Interestingly, they observed that genetically proxied PCSK9 inhibition is associated with increased direct bilirubin in European and African American populations. Although previously viewed as a potentially harmful byproduct of heme breakdown, an increasing body of evidence suggests that mildly increased serum bilirubin levels can function as protective antioxidants and anti-inflammatory agents, offering defense against the development and progression of cardiovascular disease.^10^ This aligns with the findings from a previous Mendelian randomization study that showed that individuals carrying PCSK9 loss-of-function variants show an added decrease in cardiovascular disease risk beyond the benefits of LDL reduction alone.^11^ These findings imply an additional advantage of PCSK9 inhibition for cardiovascular disease, particularly for individuals with suboptimal lipid control who are already on statin therapy. Whether it is bilirubin or other mechanisms that contribute to this added protection from PCSK9 inhibition in reducing cardiovascular events remains a question that future studies will need to address.

The study by Rosoff et al^9^ provides compelling genetic support for the clinical observations that molecules targeting apolipoprotein B catabolism carry a significantly lower risk of hepatic adverse events compared with lipid-lowering drugs that focus on apolipoprotein B secretion. Consequently, the findings presented in this study offer reassurance regarding the long-term hepatic safety of intracellular PCSK9 inhibition and support the potential use of PCSK9 inhibitors in patients with pre-existing liver disease, including the recently approved short interfering RNA inclisiran.

Overall, this timely study highlights the power of harnessing genetic and statistical approaches for adverse event profiling. Their work provides an excellent model for efficient generation of causal therapeutic evidence, which has the potential to accelerate the translation of emerging “omics” data sets into pharmacologic insights.
